# Comparative Transcriptome Analysis of Purple and Green Non-Heading Chinese Cabbage and Function Analyses of *BcTT8* Gene

**DOI:** 10.3390/genes13060988

**Published:** 2022-05-31

**Authors:** Liping Tang, Dong Xiao, Yuqin Yin, Haibin Wang, Jianjun Wang, Tongkun Liu, Xilin Hou, Ying Li

**Affiliations:** State Key Laboratory of Crop Genetics & Germplasm Enhancement, Key Laboratory of Biology and Genetic Improvement of Horticultural Crops (East China), Ministry of Agriculture and Rural Affairs of the P. R. China, Engineering Research Center of Germplasm Enhancement and Utilization of Horticultural Crops, Ministry of Education of the P. R. China, Nanjing Agricultural University, Nanjing 210095, China; 2019104058@njau.edu.cn (L.T.); dong.xiao@njau.edu.cn (D.X.); 2021104058@stu.njau.edu.cn (Y.Y.); 2020204029@stu.njau.edu.cn (H.W.); wangjianjun@njau.edu.cn (J.W.); liutk@njau.edu.cn (T.L.); hxl@njau.edu.cn (X.H.)

**Keywords:** non-heading Chinese cabbage, anthocyanin, *BcTT8*, bHLH TF, transcriptome analysis

## Abstract

Non-heading Chinese cabbage (*Brassica campestris* ssp. *chinensis*) is an important vegetative crop in the south of China. As an antioxidant, anthocyanin is the major quality trait for vegetables with purple leaves or petioles. However, the molecular biosynthetic mechanism of anthocyanin in non-heading Chinese cabbage has not been explained exclusively. In this study, two non-heading Chinese cabbage with contrasting colors in the leaves were used as the materials for RNA-seq. A total of 906 DEGs were detected, and we found that the anthocyanin and flavonoid biosynthetic pathways are significantly enriched in the purple NHCC. The transcriptome result was verified by RT-qPCR. Though bioinformatics analysis, *BcTT8* was selected as the candidate gene for the regulation of anthocyanin synthesis, and the characterization of *BcTT8* was elucidated by the functional analyses. The results proved that BcTT8 is a nucleus protein and phylogenetically close to the TT8 protein from *Brassica*. After silencing *BcTT8*, the total anthocyanin content of pTY-*BcTT8* plants decreased by 42.5%, and the relative expression levels of anthocyanin pathway genes *BcDFR*, *BcLODX* and *BcUF3GT-1* were significantly downregulated, while the transcription level of *BcFLS* was significantly upregulated. Compared with the wild type, the transgenic *Arabidopsis* showed obvious violet in the cotyledons part, and the anthocyanin biosynthetic genes such as *AtDFR* and *AtLODX* were significantly upregulated. In conclusion, *BcTT8* is critical in the anthocyanin synthesis process of non-heading Chinese cabbage. Our findings illustrated the molecular mechanism of anthocyanin biosynthesis in non-heading Chinese cabbage.

## 1. Introduction

Anthocyanins are a type of water-soluble pigment that belongs to the flavonoid family, and they play a role in plant color, development, and reactions to their biotic and abiotic environments [1]. Except for these biological functions in the plant, they are also involved in human health, since they can be employed to prevent cardiovascular and neurological illnesses [2]. However, the biosynthesis of anthocyanins is also influenced by the abiotic stress, such as temperature, high light intensity, sucrose, UV irradiation, and drought [3,4,5,6,7].

In many plant species, the anthocyanin production pathway is conserved and well elucidated [8,9,10]. There are four steps for anthocyanin biosynthesis. The first key step is that chalcone synthase (CHS) catalyzes the production of naringenin chalcone from one molecule of 4-coumaroyl CoA and three molecules of malonyl CoA. Then, the production of naringenin, dihydrokaempferol, dihydroquercetin, or dihydromyricetin is catalyzed by the chalcone isomerase (CHI), flavanone 3-hydroxylase (F3H), flavonoid 3′-hydroxylase (F3′H) and flavonoid 3′5′-hydroxylase (F3′5′H), respectively. Subsequently, dihydroflavonol-4-reductase (DFR) catalyzes the reaction to form colorless leucoanthocyanins, which are used for leucoanthocyanidin dioxygenase/anthocyanidin synthase (LODX/ANS) to produce colored anthocyanidins. Finally, the UDP-glucose flavonoid-3-*O*-glucosyltransferase (UF3GT) modifies the colored anthocyanidins to form stable anthocyanins [11].

Anthocyanin biosynthesis-related transcription factors are composed of three major types: MYB, basic helix–loop–helix (bHLH), and WD40 repeat (WDR) transcription factors [12]. The MYB family protein can be classified into four groups depending on the number of adjacent repeats: R3, R2R3, R1R2R3, 4R MYB types [12,13]. The R2R3-MYB is the largest subgroup of the MYB family involved in the flavonoid pathway. The R2R3-MYB transcription factors have an *n* terminal DNA-binding domain (the MYB domain) and an activation or repression domain usually located at the C terminus [14]. The R2R3-MYB genes *PAP1*/*MYB75*, *PAP2*/*MYB90*, *MYB113* and *MYB114* positively regulate anthocyanin synthesis [14]. In addition, bHLHs are also involved in regulating anthocyanin biosynthesis. The subgroup Ⅲf bHLH transcription factor had been reported to mediate the anthocyanin synthesis. In *Arabidopsis*, bHLH transcription factors TT8, EGL3, and GL3 are responsible for anthocyanin biosynthesis [15]. The bHLH proteins influence anthocyanin synthesis by directly activating the anthocyanin pathway genes or forming the MBW complex to mediate the structural genes. It was reported that *DcTT8* could regulate anthocyanin in *Dendrobium candidum* through inducing the expression of *DcF3′H* and *DcUFGT* [16], and the same activation pattern was also observed in other higher plants such as *Nelumbo nucifera* and tree peony (*Paeonia suffruticosa*) [17,18]. What is more, in *Arabidopsis*, MBW complexes could directly target *AtDFR* and *AtLODX* to regulate their transcriptional activity and in *Medicago truncatula* MtTT8 together with MtWD40-1, they could directly target activated promoters of *MtANS* to regulate anthocyanin synthesis [19,20]. In addition, in radish, *RsTT8* activates the *RsCHS* and *RsDFR* promoters when co-expressed with *RsMYB1* [21].

Non-heading Chinese cabbage (NHCC), which belongs to the *Brassica* family, has a great economic value in agricultural production [22]. The main secondary metabolites of non-heading Chinese cabbage includes flavonols, flavones, and anthocyanin [23]. The purple *Brassica* crops receive increased attention from the public for high levels of anthocyanin accumulation in mature leaves [24]. In the *Brassica* crop, the heterologous over-expression of *BrTT8* cloned from ‘Zi He’ (*Brassica rapa* var. *chinensis*) increased the anthocyanin content and upregulated the expression level of *CHS2*, *F3H*, and *UFGT* genes in regeneration tomato shoots [25]. Additionally, RNA-seq revealed in purple Pak-Choi (*Brassica Campestris* L. ssp. *chinensis* L. Makino) that the transcript levels of several structural genes related to anthocyanin synthesis were significantly upregulated [26]. Although there are some studies about the molecular regulation of anthocyanin in *Brassica* [27], the molecular regulatory mechanisms of non-heading Chinese cabbage have not been elucidated exclusively.

## 2. Materials and Methods

### 2.1. Plant Materials

The experimental materials were planted and located on the 15th September of 2020 in Jiangsu Agricultural Expo Park (119°01′ N, 31°09′ E). After growing for two months, we collected the leaves of purple NHCC ‘HP072′ and green NHCC ‘HG072′ to perform the experiment. Three biological replicates were quickly put in the liquid nitrogen and then sequenced by the company Biomarker (Beijing, China).

### 2.2. Determination of Total Anthocyanin Content

The total anthocyanin content was detected by the pH differential method [28]. First of all, 100 mg of fresh leaves were dipped in 3.4 mL methanol (40% acetic acid) and sonicated for 30 min; then, they were centrifuged for 10 min at 3000 rpm. All supernatants were filtered with a 0.45 μm filter. The filtrate was diluted 20 times, taken in two 1 mL samples, and reacted with 4 mL KCl (pH 1) and 4 mL NaAc (pH 4.5), respectively. After incubation for 30 min at room temperature, we measured the absorbance at 510 nm and 700 nm in an Enzyme Linked Immunosorbent Assay (ELISA) and calculated the total anthocyanin content.

### 2.3. Transcriptome Analysis of Green and Purple NHCC

An mRNA isolation kit was utilized to isolate the total mRNA from the leaves (Aidlab, Beijing, China). The quality of mRNA concentration was measured using a NanoDrop 2000 (Thermo Fisher Scientific, Wilmington, DE, USA). The NEBNext UltraTM RNA Library Prep Kit for Illumina was used (NEB, Ipswich, MA, USA) to generate the sequencing libraries. The Illumina HiSeq2500 platform (San Diego, CA, USA) at Biomarker (Beijing, China) was applied to sequence the libraries. The FPKM (fragments per kilobase of transcript per million fragments mapped) method was applied to calculate the gene expression levels. The NHCC001 genome was used as the reference genome [29].

### 2.4. Gene Function Annotation and Differential Expressed Gene Identification

In this study, we exploited the six common databases to access the gene function annotation: Nr (NCBI non-redundant protein sequences, RefSeq non-redundant proteins (nih.gov)); Nt (NCBI non-redundant nucleotide sequences); Pfam (http://pfam.xfam.org/ (accessed on 25 January 2020)); KOG/COG (http://www.ncbi.nlm.nih.gov/COG/ (accessed on 1 February 2020)); Swiss-Prot (http://www.expasy.ch/sprot (accessed on 7 February 2020)), KO (http://www.genome.jp/kegg/ (accessed on 12 February 2020)); GO (Gene Ontology, http://www.geneontology.org/ (accessed on 13 February 2020)). We took use of the DESeq2 to analyze the differential expression genes of two groups [30]. We used Benjamini and Hochberg’s approach for controlling the false discovery rate (FDR) (*p* < 0.05). The differentially expressed genes (DEGs) were selected on the basis of having at least a two-fold difference in expression between the HG072 and HP072 (*p* < 0.05). GO analysis of the DEGs was carried out using the topGO package (*p* < 0.05).

### 2.5. Expression of Anthocyanin-Related Pathway Genes in Green and Purple NHCC

To verify the results of transcriptome analysis, RT-qPCR was carried out for anthocyanin pathway genes. The reverse transcription of mRNA was used the Evo M-MLV RT Kit II (Accurate Biotechnology, Hunan, China) as directed by the protocols. RT-qPCR was carried out on the ABI StepOne (Applied Biosystems, Waltham, MA, USA) with Hieff^®^ qPCR SYBR Green Master Mix (Yeasen, Shanghai, China) in triplicate. Data were normalized with *BcACTIN* gene of NHCC, and the 2^−ΔΔCT^ method was employed for analysis [31]. The gene-specific primer sequences are listed in Supplementary Table S1.

### 2.6. Sequence Analysis of BcTT8

The coding sequence of *BcTT8* gene from the transcriptome result was blasted in the NHCC database (http://nhccbase.njau.edu.cn/website/ (accessed on 1 June 2020)). We identified the coding sequence of *BcTT8* through ORF Finder (https://www.ncbi.nlm.nih.gov/orffinder/ (accessed on 3 June 2020)) The structure analysis of BcTT8 protein was identified through the online website NCBI-CDD (https://www.ncbi.nlm.nih.gov/Structure/cdd/wrpsb.cgi (accessed on 5 June 2020)). Homologous sequences of other species were found by NCBI-BLAST (https://blast.ncbi.nlm.nih.gov/Blast.cgi (accessed on 5 June 2020)). The sequences alignments were carried out by DNAMAN 9.

### 2.7. Subcellular Localization Assays and Analysis of Phylogenetic Tree

We amplified the coding sequence of *BcTT8* using the gene-specific primers (Supplementary Table S1) and then cloned it into the PRI101 vector with a *CaMV35S* promoter. The construct was transformed into *A*. *tumefaciens* strain GV3101, and we resuspended the overnight cultures of *A*. *tumefaciens* strains with infiltration buffer (10 mM MgCl_2_, 10 mM MES, and 0.1 mM acetosyringone) to OD_600_ at 0.8 and incubated them at room temperature for 4 h. The suspension was infiltrated into *Nicotiana. benthamiana* leaves. The injected plants were grown under the appropriate growth condition for about 60 h; next, the leaf samples were observed using the Laser Scanning Confocal Microscope (Zeiss LSM780); *35S*:GFP alone served as the control. A neighbor-joining phylogenetic tree was constructed with MEGA X (1000 bootstrap replicates).

### 2.8. Silencing of BcTT8 through VIGS System

To silence of *BcTT8*, we designed a self-hybridizing palindromic oligonucleotide of 80 nt (Supplementary Table S1) following the protocol [32]. The primers p-TYMV-F and p-TYMV-R were used to identify the pTY-*BcTT8* plasmid with the expected size (1566 nt). The total of 50μg purified pTY-*BcTT8* plasmid was diluted with 50μL ddH_2_O; then, we mixed the plasmid with 0.1 M spermidine, 10 μL gold power and 0.1 M CaCl_2_ in the 2 mL tubes on ice for 20 min. The mixture was centrifuged at 12,000 rpm for 15 s, and it was washed 4 times using the ethanol (100%). For infecting, we utilized the particle bombardment, and the empty VIGS vector (pTY-S) plasmid was inoculated as a control.

### 2.9. Overexpression of BcTT8 in Arabidopsis

The coding sequence of *BcTT8* was cloned into vector PRI101-GFP; *BcTT8*-GFP plasmid was transformed into *Agrobacterium tumefaciens* strain GV3101 and cultured in LB liquid medium with antibiotics (50 mg·L^−1^ kanamycin and 50 mg·L^−1^ rifampicin). We conducted this experiment by the floral dip method [33]. Overnight cultures of *A. tumefaciens* strains were resuspended and diluted using the 5% sucrose solution buffer (pH 5.8) containing 0.01–0.05% (vol/vol) Silwet L-77 to OD_600_ ≈0.8. Then, we dipped the *Arabidopsis* inflorescences for 60 s until the resuspended *Agrobacterium* cells carrying the *BcTT8* gene were transferred. To obtain the transformants, the treated plants were selected with the solid medium with 50 mg·L^−1^ kanamycin and 160 mg·L^−1^ timentin.

### 2.10. Statistical Analysis

We analyzed the data through Microsoft Excel 2021 and the statistical significance of the differences between the two cultivars was determined with by an unpaired t-test with SPSS 22.0. Significant differences (*p* < 0.05) were indicated with different letters.

## 3. Results

### 3.1. Samples Expression Pattern and Differentially Expressed Genes Clustering

**Figure 1:**
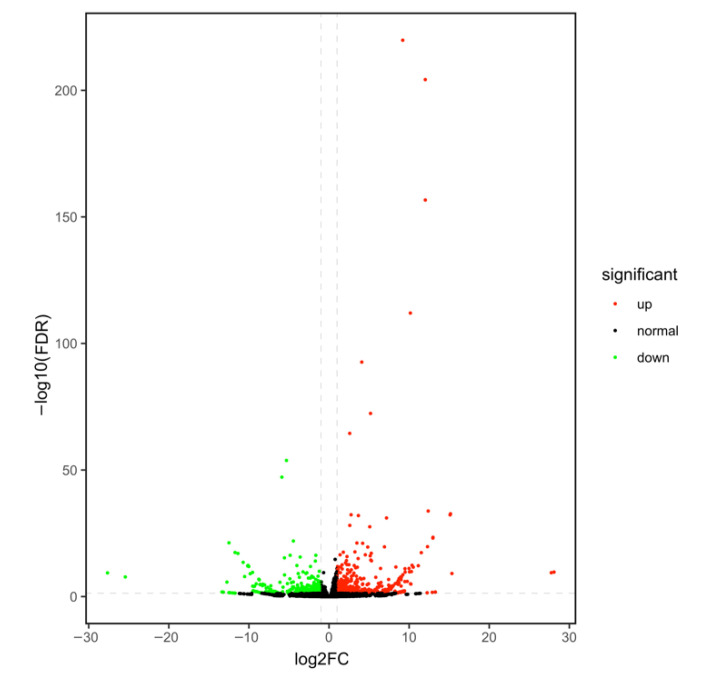
The volcano map tells us the expression trends of these DEGs between green and purple samples; the red dots and green dots present the upregulation and downregulation of DEGs, respectively, while the black dots mean genes without a significant difference in expression between the two samples.

In our study, we measured the total anthocyanin content in the two non-heading Chinese cabbage varieties; the total anthocyanin content of purple NHCC is 3.5 folds higher than the green one, which is 7.57 mg·100 g^−1^ and 2.26 mg·100 g^−1^, respectively (Figure S1). Based on the anthocyanin difference between the two cultivars, we performed comparative transcriptome analysis. The sequencing results contained a total of 906 DEGs, of which 520 DEGs showed upregulation and 386 DEGs showed downregulation (Figure 1 and Supplementary Table S2) Among these, we annotated 11 classes of transcription factor family protein, and the bHLH family was comprised four genes, of which only *BcTT8* was upregulated (Table 1 and Supplementary Table S3).

### 3.2. Differentially Expressed Genes GO Enrichment

**Figure 2:**
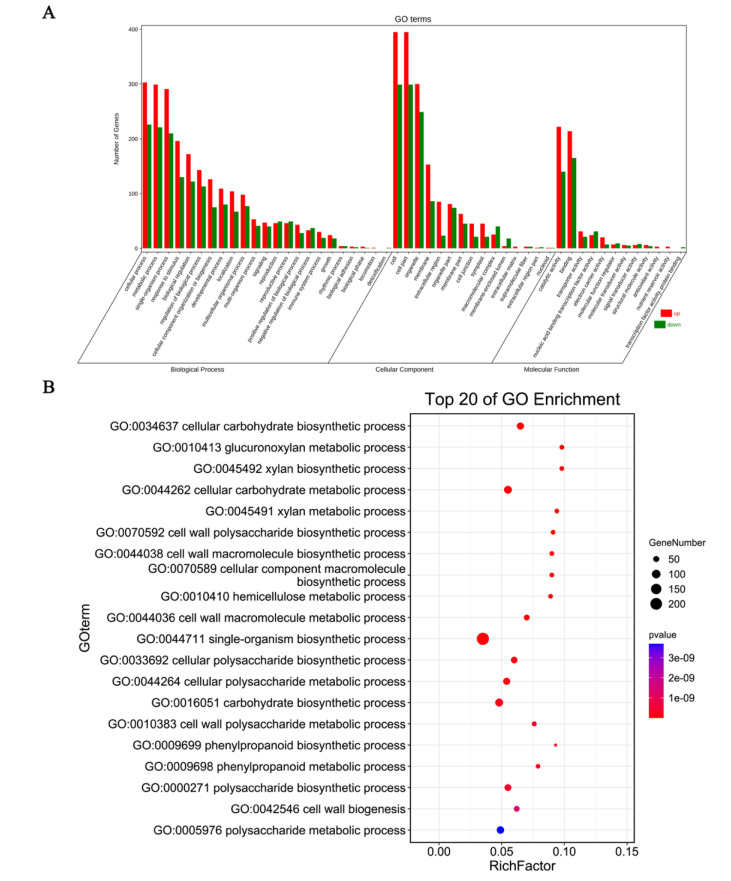
Gene Ontology classification enrichment analysis results of DEGs. GO classification of upregulated and downregulated DEGs (**A**), the Top 20 GO enrichment process (**B**). Rich Factor: DEGs numbers/total gene numbers enriched in the process.

In our result, the DEGs genes were enriched in the GO terms and further classified into three categories: the cell component category, biological process as well as molecular function process (Figure 2A and Supplementary Table S4). A total of 377 upregulated and 271 downregulated unigenes were annotated to GO terms in the biological process, of which most of the DEGs were mainly linked to the metabolic process, cellular process and single-organism process (Supplementary Table S5). A total of 754 DEGs were annotated into the cell component category, including 441 upregulated and 313 downregulated genes. For the category of cell component, most of the upregulated and downregulated unigenes were further classified into cell, cell part, and organelle terms (Supplementary Table S6). In the molecular function process, a total of 581 DEGs were enriched into this classification, and most of them were mainly related to the catalytic activity and binding terms (Supplementary Table S7). 

For the GO functional enrichment, the top 20 GO functional process was annotated (Figure 2B). The ‘anthocyanin-containing compound biosynthetic’ process (GO:0009718) was not in the top20 GO biological terms, while it was also significantly enriched (2.71 × 10^−6^, *p* < 0.05) (Supplementary Table S8). A total of 15 DEGs involved in the ‘anthocyanin-containing compound biosynthetic’ process and the upregulated DEGs were comprised of the anthocyanin accumulation genes *BcCHI-1*, *BcCHI-2*, *BcDFR*, *BcLODX*, *BcUF3GT-1*, *BcUF3GT-2*, *BcUF75C1*, *BcTT19-1*, *BcTT19-2*, *Bc5MAT* and transcription factors BcTT8, BcMYBL2-1, and BcMYBL2-2 (Table 2).

### 3.3. Differentially Expressed Genes KEGG Enrichment

**Figure 3:**
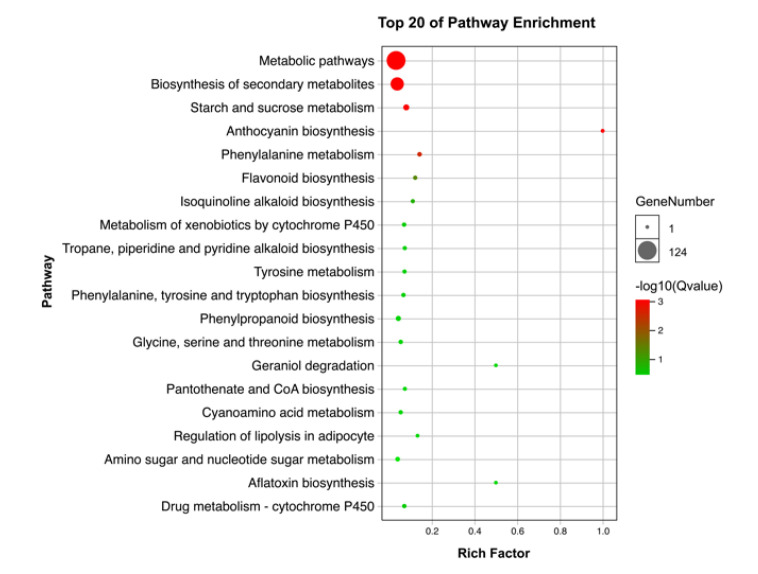
The 20 most KEGG pathway enrichment for DEGs. Rich Factor: DEGs numbers/total gene numbers enriched in the pathway.

We performed the KEGG pathway enrichment to annotate the key genes of the anthocyanin biosynthesis pathway, and a total of 245 unigenes were identified (Supplementary Table S9). The 20 most KEGG pathways are shown (Figure 3). In the anthocyanin biosynthetic pathway (ko00942), *BcUF3GT-1*, *BcUF3GT-2*, and *BcUF75C1* were detected, which could encode the UDP-glucose flavonoid-3-O-glucosyltransferase transferase protein (Table 3). Six DEGs were enriched in the flavonoid biosynthetic pathway (ko00941), including *BcCHI-1*, *BcCHI-2*, *BcDFR*, *BcLODX*, *BcFLS*, and *BcC4H* (Table 3). There were no *BcCHS*, *BcF3H* and *BcF3′H* in the list, but several structural genes *BcCHI-1*, *BcCHI-2*, *BcDFR*, *BcLODX, BcUF3GT-1*, *BcUF3GT-2,* and *BcUF75C1* were involved in the anthocyanin biosynthesis pathway, showing the upregulation.

### 3.4. Verification of Transcriptome Result by RT-qPCR

**Figure 4:**
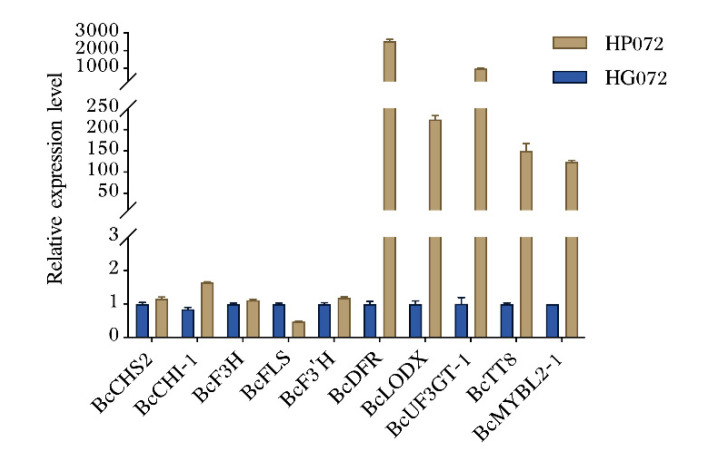
Analysis of genes related to anthocyanin biosynthesis-related genes using the RT-qPCR. Gene expression levels were normalized to *BcActin*. Error bars represent the standard error of the mean (*n* = 3).

In order to verify the results, several genes related to anthocyanin biosynthesis were selected and measured by RT-qPCR (Figure 4). The result indicated that the transcript expression levels of *BcDFR* (*BraC09g018850*), *BcLODX* (*BraC03g052160*) and *BcUF3GT-1* (*BraC06g022480*) in HP072 were remarkably more upregulated than those in HG072. Similarly, the relative expression levels of transcription factors BcTT8 (BraC09g027820) and BcMYBL2-1 (BraC07g035800) were also significantly higher in HP072 than in HG072. However, the expression levels of the early anthocyanin biosynthesis genes (EBGs) *BcCHS2* (*BraC10g026540*), *BcF3H* (*BraC02g029180*) and *BcF3′H* (*BraC08g015770*) showed no difference between these two samples (Table 4). The relative expression levels of these genes were consistent with the transcriptome analysis result.

### 3.5. Characterization and Phylogenetic Analysis of BcTT8

**Figure 5:**
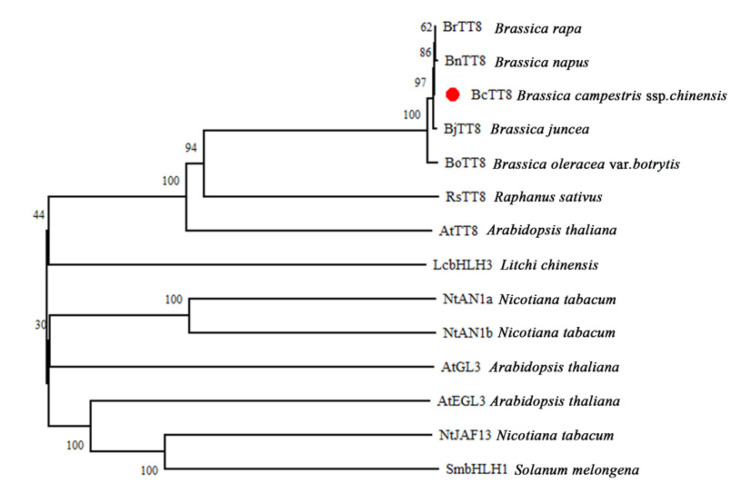
The phylogenetic tree of BcTT8 protein in non-heading Chinese cabbage and similar bHLH proteins in other species. The protein labeled with a red dot was BcTT8. Gene bank number BrTT8 (XP_009113574.1); BjTT8 (AIN41653.1); RsTT8 (ASF79354.1); BnTT8 (QFU95692.1); BoTT8 (ADP76654.1); NtAN1a (NP_001312042.1); NtAN1b (NP_001289454.1), AtGL3 (NP_680372); AtEGL3 (NP_176552); AtTT8 (CAC14865); SmbHLH1 (AFJ05597.1); LcbHLH3 (APP94124.1).

The *BcTT8* homologous clone result showed that it encodes a 1566 bp nucleotide sequence and the ORF encodes a full function protein with 521 amino acids. Structure analysis results demonstrated that BcTT8 belongs to the bHLH family, which contains the conserved bHLH-MYC-N and the bHLH superfamily domains (Figure S2). Multiple sequences analysis for BcTT8 and other homologous proteins (Figure S3). A phylogenetic tree was performed to analyze the homologous relationship between BcTT8 and similar bHLH proteins in other species. The result showed that BcTT8 had the closest phylogeny with BoTT8 (*Brassica oleracea* var. *botrytis*) (Figure 5).

### 3.6. Subcellular Localization of BcTT8

**Figure 6:**
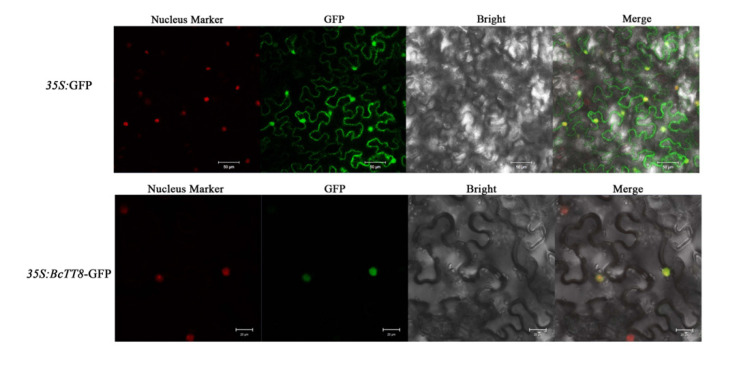
Subcellular localization of *35S*:GFP and *35S*:*BcTT8*-GFP, bars = 20 μm, *35S*:GFP was used as a control.

We constructed a *35S*:*BcTT8*-GFP fusion vector to analyze the subcellular localization of BcTT8 protein. The suspension was infiltrated into *N. benthamiana* leaves. In the cell nucleus, we observed the *BcTT8*-GFP fusion protein while the empty vector GFP protein was observed in both the nucleus and the cytoplasm, which indicated the BcTT8 functions in the cell nucleus (Figure 6).

### 3.7. Expression Analysis of Structural Genes after Silencing of BcTT8

**Figure 7:**
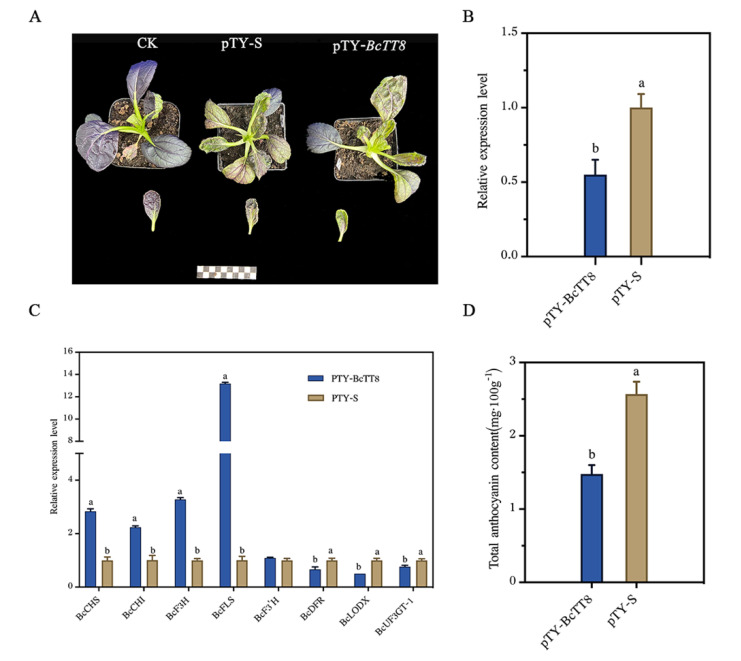
Photographs of WT, pTY-S and pTY-*BcTT8* plants (**A**) and RT-qPCR analysis result of *BcTT8* and anthocyanin pathway genes (**B**,**C**) and total anthocyanin content in plants (**D**). Significant differences (*p* < 0.05) were indicated with different letters.

In this study, we obtained from these plants emerged color fading, which was one of the viral symptoms. However, the color variations among control plants, infected pTY-S plasmid plants and the infected pTY-*BcTT8* plants were obviously different. Both the viral plants appeared to have color fading, but the one inoculated with pTY-*BcTT8* presented barely violet (Figure 7A), and the silencing efficiency of *BcTT8* expression was about 50% compared with control (Figure 7B). We performed the RT-qPCR assay for analyzing the transcription expression levels of anthocyanin synthesis-related genes. The expression levels of *BcCHS*, *BcCHI* and *BcF3H* were significantly increased in pTY-*BcTT8* plants, while *BcF3′H* showed no difference between pTY-S and pTY-*BcTT8* plants. *FLS* (flavonol synthase) is regarded as the key gene for the biosynthesis of flavonols, and in the present study, the *BcFLS* showed significantly high expression in pTY-*BcTT8* plants. The expression levels of *BcDFR*, *BcLODX* and *BcUFG3T-1* were significantly declined in pTY-*BcTT8* plants compared with pTY-S plants (Figure 7C).

We determined the total amount of anthocyanin content; the content of pTY-*BcTT8* silencing plants was about 57.5% for the content of pTY-S plants, which was 1.48 mg·100 g^−1^ and 2.57 mg·100 g^−1^, respectively (Figure 7D). We proposed that the silencing of *BcTT8* caused the redirection of metabolism flux to flavonol synthase that reduced the anthocyanin accumulation.

### 3.8. Heterologous Expression Analysis of BcTT8 in Arabidopsis

**Figure 8:**
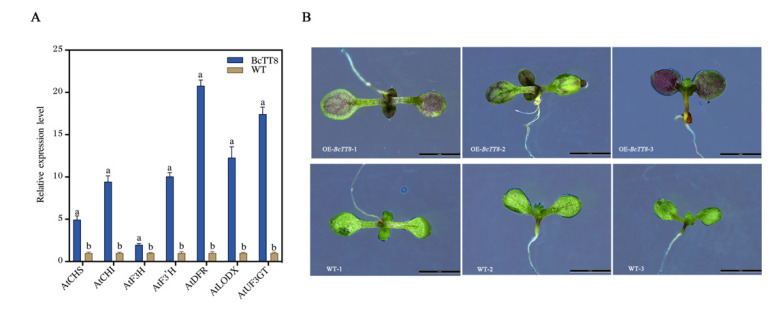
Identification of *BcTT8* transgenic plants. RT-qPCR analysis of anthocyanin pathway genes in *BcTT8*-overexpression plants (**A**) and photographs of WT and *BcTT8* transgenic plants taken by stereoscopic microscope, bars = 5 mm (**B**).

In order to elucidate the function of *BcTT*8, we constructed a *35S*:*BcTT8* vector using an *Agrobacterium*-mediated floral dip method. The coding sequence of *BcTT8* was 1566 bp, and three transgenic plants were selected from the MS solid medium (Figure S4). Compared with wild-type plants, *BcTT8*-overexpressed plants had increased transcription levels of anthocyanin biosynthesis pathway genes. *AtCHS*, *AtCHI*, *AtF3H*, *AtF3′H*, *AtDFR*, *AtLODX*, and *AtUF3GT* were all significantly upregulated (Figure 8A). The cotyledons of transgenic plants presented obviously violet, but the WT still appeared green (Figure 8B), which demonstrated that *BcTT8* promoted anthocyanin synthesis in *Arabidopsis*.

## 4. Discussion

Transcriptome analysis is a powerful tool for selecting the differentially expressed genes (DEGs) with our samples, which are useful to find the candidate genes. Contrasting transcriptome analysis had been performed in two Pak-Choi, and they found that in the purple variety, ‘flavonoid biosynthesis’ was the only KEGG significantly enriched pathway, which comprises structural genes *BrDFR*, *BrLODX*, *BrUF3GT-1*, *BrUF3GT-2*, and *BrUF75C1* [26]. As for the release of the NHCC001 genome [29], we identified several enriched anthocyanin-related pathways and further explain the mechanism of anthocyanin regulation. Our results have many differences with the previous studies, except for the ‘flavonoid’ pathway, the ‘anthocyanin biosynthesis’, ‘starch and sucrose metabolism’, and ‘biosynthesis of secondary metabolites’ pathways, which were also significantly enriched (Figure 3). Phenylpropane and flavonoid pathway genes participate in synthesizing the precursors of anthocyanin, which is also a subgroup of flavonoid [15], so that genes that participate in the ‘flavonoid’ and ‘phenylalanine metabolism’ pathways were significantly enriched. What is more, catalyzing anthocyanin synthesis requires ample enzymes, and encoding these products costs a large amount of energy by starch hydrolysis [34]; thus, it makes sense that the ‘starch and sucrose metabolism’ pathway genes were significantly enriched in the purple NHCC HG072. The KEGG pathway enrichment results confirmed that several structural genes, *BcDFR*, *BcLODX*, *BcUF3GT-1*, *BcUF3GT-2*, and *BcUF75C1*, which are related to the flavonoid and anthocyanin pathways, showed significantly expression in purple leaves (Table 3). Our analyses are in accordance with the transcriptome profiling in Pak-Choi and red Chinese cabbage (*Brassica Rapa*), of which the *BrDFR*, *BrLDOX*, *BrUF3GT*, and *BrUGT75C1-1* are highly expressed [26,35], and relevant studies have revealed that these genes are critical in the process of anthocyanin biosynthesis [36].

Utilizing comparative RNA sequencing, researchers found that MYB and bHLH TFs are involved in the anthocyanin biosynthetic pathway [37,38]. MYB and bHLH TFs could finely tune the expression of anthocyanin pathway genes, so it is crucial to analyze transcription factor expression levels that could provide thorough insights into the regulatory mechanism of anthocyanin synthesis. In our study, we identified that *BcTT8* was more significantly expressed in purple leaves (Figure 4), indicating that *BcTT8* functions as an anthocyanin biosynthetic regulator. Earlier studies reported that *NnTT8* recovered anthocyanin accumulation in *Arabidopsis tt8* mutant [18], and other bHLH family proteins were also proved to regulate anthocyanin biosynthesis in other higher plants [39,40,41]. In our study, both the pTY plants and pTY-*BcTT8* plants presented color fading (Figure 7A), which was a symptom of virus injection [42], but the silencing of *BcTT8* led to more severe symptoms. *BcTT8*-silenced non-heading Chinese cabbage showed a notable downregulation of anthocyanin biosynthetic genes *BcDFR*, *BcLODX*, and *BcUF3GT*, while the transcription level of *BcFLS* increased considerably (Figure 7C). Previous studies had proved that in other plant species, bHLH transcription factors could activate the expression of *DFR*, *ANS,* and *UFGT*, which improve the anthocyanin content [16,17]; thus, we proposed that in non-heading Chinese cabbage, transcription factor BcTT8 also facilitates the similar function, which could explain the downregulation of the LBGs and the decrease in anthocyanin production. We should mention that the production of flavonols and anthocyanins share the same biosynthesis pathway and compete for the same precursors. Flavonol synthase (FLS) may direct the dihydroflavonol precursors to the flavonol route [8]. In our study, *BcFLS* exhibited significant upregulation in the *BcTT8*-silencing plants as the anthocyanin content decreased dramatically. The metabolic flux redirection was also observed in other higher plants. Mutations in *ScbHLH17* prevented the biosynthesis of anthocyanins in white *Seneclo cruentus* cultivars, and the RNAi silencing lines of anthocyanidin reductase (ANR) induced a redirection of the proanthocyanidin as well as the flavonol biosynthesis pathway, causing a reduction in anthocyanin synthesis in strawberry [43,44].

In *Caryophyllales* plants, the suppression of *DFR* and *ANS* resulted in the lack of anthocyanin, but the ectopic overexpression of these two genes induced anthocyanin accumulation [45]. In the *BcTT8*-overexpressed lines, the relative expression levels of anthocyanin structural genes showed significant upregulation, especially the LBGs *AtDFR*, *AtLODX* and *AtUF3GT*, causing the transgenic plants to present obviously violet (Figure 8A,B).

Brassicaceous vegetables have been receiving scientific attention for many years because numerous studies reported that eating these vegetables would reduce the risk of some chronical diseases and kinds of cancer [46,47]. The main reason for that is that brassicaceous vegetables contain various phytonutrients such as the polyphenol, glucosinolates, carotenoid or terpenoid groups. Currently, purple brassicaceous vegetables, including non-heading Chinese cabbage, Chinese cabbage, Zicaitai, and kale have become increasingly popular not only for their attractive colors but also for the benefits they bring to the public. An increasing number of studies have pointed out that diets in anthocyanins help lower the risk of cancer, cardiovascular diseases, diabetes, oxidative stress, inflammation, and related diseases [48,49,50]. Non-heading Chinese cabbage is a nutrition-rich vegetable that is widely consumed worldwide, but the molecular mechanism of anthocyanin synthesis is under explored. In this study, we identified that *BcCHI-1*, *BcCHI-2*, *BcDFR*, *BcLODX*, *BcUF3GT-1*, *BcUF3GT-2*, *BcUF75C1,* and one bHLH transcription factor BcTT8 were significantly upregulated in purple NHCC, and functional analyses demonstrated that *BcTT8* could positively promote anthocyanin accumulation. Our findings illustrated the anthocyanin molecular regulation of non-heading Chinese cabbage, which could provide the theoretical basis for breeding high anthocyanin content non-heading Chinese cabbage cultivars.

## 5. Conclusions

In the present study, we have a further understanding of the anthocyanin biosynthetic pathway in non-heading Chinese cabbage through the comparative transcriptome analysis. A number of DEGs related to anthocyanin and flavonoid biosynthesis pathways were identified, indicating their important roles in the anthocyanin biosynthesis in NHCC. In addition, we explained the function of *BcTT8* gene and demonstrated that *BcTT8* is of great importance in anthocyanin synthesis.

## Figures and Tables

**Table 1:** DEGs of bHLH Gene Family.

Gene Name	Gene ID	Mean FPKM (Purple)	Mean FPKM (Green)	Log2FC
*BcTT8*	BraC09g027820	20.971186	0.128603	7.1793481
*BcEGL1*	BraC09g014020	0.164681	0.77609633	−2.7202394
*BcGL3*	BraC04g016160	0.374962	1.52723467	−1.6471003
*BcPRE1*	BraC07g019880	1.330022	6.18879667	−2.371988

**Table 2:** Anthocyanin-Containing Compound Biosynthetic Process (qvalue = 2.71 × 10^−6^).

Gene Name	Gene ID	Mean FPKM (Purple)	Mean FPKM (Green)	Log2FC	Up or Down Regulated
*BcCHI-1*	BraC09g053560	71.52371067	30.162151	1.4134447	up
*BcCHI-2*	BraC09g053860	49.64995433	19.18305767	1.54414961	up
*BcDFR*	BraC09g018850	332.763204	0.084531	12.0172491	up
*BcLODX*	BraC03g052160	35.953383	0.135272333	8.22759096	up
*BcNCED4*	BraC08g01423	0	0.688357333	−8.7651817	down
*BcFLS*	BraC10g030090	107.904424	259.9946647	−1.1015793	down
*BcUF3GT-1*	BraC06g022480	205.7734173	0.050154333	12.021179	up
*BcUF3GT-2*	BraC10g012540	57.12839133	0	15.1169748	up
*BcUF75C1*	BraC08g010530	322.273295	0.611051	9.20124194	up
*BcTT19-1*	BraC02g007050	82.251634	10.887416	3.10472421	up
*BcTT19-2*	BraC10g024210	90.53810367	0.047466333	11.1466138	up
*Bc5MAT*	BraC09g003150	83.30002233	0.080537667	10.1547666	up
*BcTT8*	BraC09g027820	16.56795833	0.128603	7.1793481	up
*BcMYBL2-1*	BraC07g035800	42.067511	1.699416333	4.83412855	up
*BcMYBL2-2*	BraC02g021000	14.61018833	3.159260333	2.38862003	up

**Table 3:** Anthocyanin and Flavonoid Biosynthesis Pathway DEGs.

Pathway	Gene Name	Gene ID	Mean FPKM (Purple)	Mean FPKM (Green)	Log2FC	Up or Downregulated
Flavonoid Biosynthesis	*BcCHI-1*	BraC09g053560	71.52371067	30.162151	1.4134447	up
	*BcCHI-2*	BraC09g053860	49.64995433	19.18305767	1.54414961	up
	*BcDFR*	BraC09g018850	332.763204	0.084531	12.0172491	up
	*BcLODX*	BraC03g052160	35.953383	0.135272333	8.22759096	up
	*BcC4H*	BraC03g016590	156.2112757	18.594747	3.233418596	up
	*BcFLS*	BraC10g030090	107.904424	259.9946647	−1.1015793	down
Anthocyanin biosynthesis	*BcUF3GT-1*	BraC06g022480	205.7734173	0.050154333	12.021179	up
	*BcUF3GT-2*	BraC10g012540	57.12839133	0	15.1169748	up
	*BcUF75C1*	BraC08g010530	322.273295	0.611051	9.20124194	up

**Table 4:** Gene list verified by RT-qPCR.

Gene Name	Gene ID	Mean FPKM (Purple)	Mean FPKM (Green)	Log2FC	Up or Down Regulated
*BcCHS2*	BraC10g026540	471.590159	471.7846887	0.000594985	
*BcCHI-1*	BraC09g053560	71.52371067	30.162151	1.4134447	up
*BcF3H*	BraC02g029180	4.807456333	7.09875	−0.525317861	
*BcF3′H*	BraC08g015770	24.735745	30.335388	−0.13065833	
*BcDFR*	BraC09g018850	332.763204	0.084531	12.0172491	up
*BcLODX*	BraC03g052160	35.953383	0.135272333	8.22759096	up
*BcFLS*	BraC10g030090	107.904424	259.9946647	−1.1015793	down
*BcUF3GT-1*	BraC06g022480	205.7734173	0.050154333	12.021179	up
*BcTT8*	BraC09g027820	16.56795833	0.128603	7.1793481	up
*BcMYBL2-1*	BraC07g035800	42.067511	1.699416333	4.83412855	up

## Data Availability

The raw data of the transcriptome have been uploaded to NCBI-Sequence Read Archive (https://www.ncbi.nlm.nih.gov/search/all/?term=SRA (accessed on 8 April 2022)). HG072-1: SRR18693103; HG072-2: SRR18691869; HG072-3: SRR18693073; HP072-1: SRR18693245; HP072-2: SRR18729142; HP072-3: SRR18693566.

## Supplementary Materials

The following supporting information can be downloaded at: https://www.mdpi.com/article/10.3390/genes13060988/s1. Figure S1: Photographs of green NHCC ‘HG072′ and purple NHCC ‘HP072′ (A) and total anthocyanin content of these two cultivars (B); Figure S2: Conversed domains analysis of BcTT8; Figure S3: Amino acids sequences blast of bHLH proteins; Figure S4: Agarose gel electrophoresis picture of *BcTT8* transgenic plants verification by RT-PCR. Table S1: Primer pairs used in this study; Table S2: All the DEGs from the transcriptome result; Table S3: Differentially expressed transcription factors list from the transcriptome result; Table S4: Classification of GO terms; Table S5: GO classification of biological process; Table S6: GO classification of cell component; Table S7: GO classification of molecular function; Table S8: Result of GO enrichment annotation: Table S9: Result of KEGG pathway annotation.
