# Supplementary figures and images for: Comparative Transcriptome Analysis of Purple and Green Non-Heading Chinese Cabbage and Function Analyses of BcTT8 Gene
Source: Genes (Basel). 2022 May 31;13(6):988. doi: 10.3390/genes13060988 (PMC9222865; doi:10.3390/genes13060988)

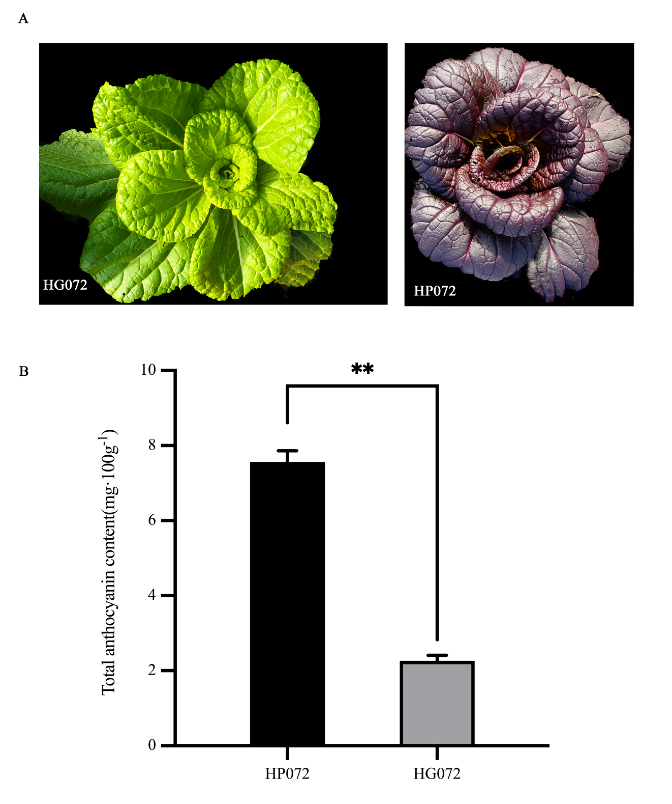

Supplement: Supplementary file 1 [file genes-13-00988-s001.zip › Supplementary figures/Figure S1.png]

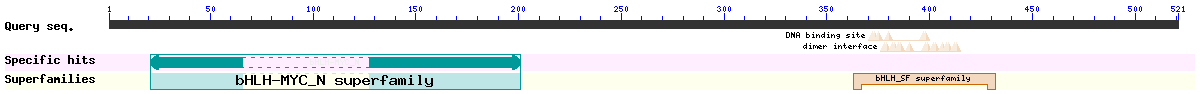

Supplement: Supplementary file 1 [file genes-13-00988-s001.zip › Supplementary figures/Figure S2.png]

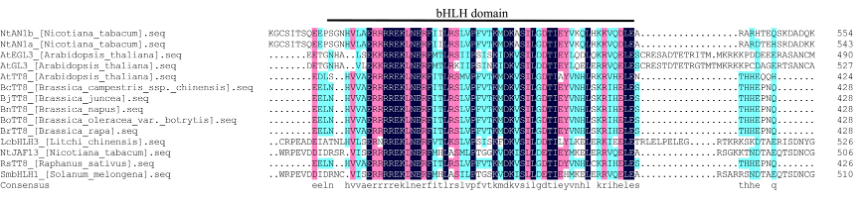

Supplement: Supplementary file 1 [file genes-13-00988-s001.zip › Supplementary figures/Figure S3.png]

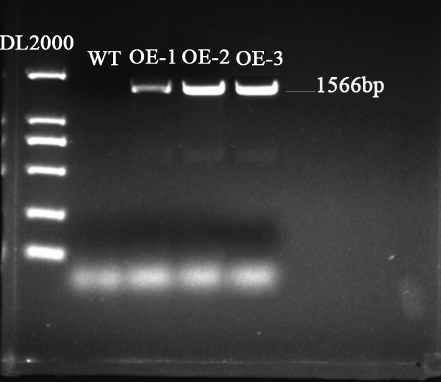

Supplement: Supplementary file 1 [file genes-13-00988-s001.zip › Supplementary figures/Figure S4 .png]
